# Chemical Profiles and Foraging Efficiency in a Carnivorous Plant: Effects of Contrasting Precipitation Regimes

**DOI:** 10.1007/s10886-026-01701-x

**Published:** 2026-02-26

**Authors:** Alessio Tei, Carla Vázquez-González, Gregory Röder, Irene Virseda, Lucía Martín-Cacheda, Sergio Rasmann, Xoaquín Moreira

**Affiliations:** 1 Misión Biológica de Galicia (MBG-CSIC), Apartado de Correos 28, Pontevedra, Galicia, 36080 Spain; 2 Institute of BioEconomy, National Research Council of Italy, Via Pietro Gobetti 101, Bologna, 40129 Italy; 3 Institute of Biology, University of Neuchâtel, Rue Emile-Argand 11, Neuchâtel, 2000 Switzerland

**Keywords:** Drosera rotundifolia, Environmental plasticity, Insect preys, North-western Spain, Semi-volatile organic compounds, Volatile organic compounds

## Abstract

Carnivorous plants have evolved specialized adaptations that allow them to persist in nutrient-poor habitats, including modified traps, digestive enzymes, and mechanisms for absorbing nutrients derived from prey. Beyond these structural features, chemical signalling mediated by volatile organic compounds (VOCs) and semi-volatile organic compounds (SVOCs) contributes to several ecological functions, such as prey attraction, short-range interactions, and defence. VOCs can attract insects over relatively long distances, whereas SVOCs tend to remain on trap surfaces, where they may influence local interactions with arthropods and microbes. Environmental conditions, particularly precipitation and humidity, are known to affect VOC emissions and may alter foraging dynamics, yet the extent to which variation in chemical emissions corresponds with differences in prey capture is still not well resolved. To address this knowledge gap, we performed a study under both field and greenhouse conditions using the carnivorous plant *Drosera rotundifolia* in two climatically contrasting sites of north-western Spain: the wetter Serra do Cando (CP) and the drier Serra de Ancares (AP). At both sites, we quantified insect prey capture and characterized VOC and SVOC emissions. Prey capture rates were similar between regions, but plants from the drier site showed higher total VOC emissions, while SVOC production did not differ markedly. PERMANOVA analyses further indicated that site had no significant effect on overall VOC or SVOC composition. Together, these results suggest a balance between flexibility in emission intensity and stability in chemical composition, providing insight into how specialized metabolites support the ecological functioning of carnivorous plants across contrasting environmental conditions.

**Supplementary Information:**

The online version contains supplementary material available at 10.1007/s10886-026-01701-x.

## Introduction

Carnivorous plants represent one of the most striking examples of evolutionary innovation, having evolved a unique set of functional traits that enable survival and reproduction in nutrient-deficient environments (Pavlovič et al. 2016; Wheeler and Carstens 2018). These habitats—often acidic, waterlogged, or composed of sandy soils—are particularly low in essential macronutrients like nitrogen and phosphorus, making conventional root-based nutrient uptake highly insufficient (Adamec 2011; Ellison and Gotelli 2002; Givnish et al. 1984). In response to these constraints, carnivorous plants have developed a suite of adaptations known as the carnivorous syndrome (Adamec et al. 2021). This includes specialized morphological structures for prey capture, such as pitfall, flypaper, or snap traps, the production of digestive enzymes, and physiological mechanisms for absorbing and assimilating nutrients from animal prey (Juniper et al. 1989; Król et al. 2012; Pavlovič and Saganová 2015; Thorén et al. 2003).

Yet, beyond this foundational syndrome, carnivorous plants show considerable morphological and functional diversity across lineages (Ellison and Gotelli 2009; Pavlovič 2025). Differences in leaf architecture, trichome density, mucilage chemistry, and trap surface properties can affect prey selectivity, capture efficiency, and overall nutrient gain (Krueger et al. 2020; Mithöfer 2022). While much is known about the structural adaptations of carnivorous plants, the role of chemical signalling in prey attraction has only recently come into focus (Hatcher et al. 2020; Pavlovič 2025). In several genera, including *Nepenthes* and *Sarracenia*, volatile organic compounds (VOCs) emitted from carnivorous plants have been shown to mimic floral or decompositional cues to attract insects (Di Giusto et al. 2010; Dupont et al. 2023; Jürgens et al. 2009). These small, airborne molecules can act over long distances, enhancing foraging efficiency in nutrient-poor habitats. In contrast, semi-volatile organic compounds (SVOCs) are more likely to condense and to accumulate on trap surfaces (Ojeda et al. 2021). Though less studied, SVOCs are thought to aid in short-range attraction, contact signalling, antimicrobial defence, and mucilage function (Hatcher et al. 2020), contributing to the multifaceted strategies carnivorous plants use to secure essential nutrients. Despite these insights, studies directly linking VOC emissions to prey capture remain scarce, limiting our understanding of their full ecological role. In one of the few available studies, Dupont et al. (2023) found that in *Sarracenia × leucophylla*, fatty-acid derivatives explained most of the variation in ant captures, whereas monoterpenes accounted for most of the variation in captures of bees, moths, Diptera, and wasps.

Abiotic factors, including precipitation, strongly influence plant physiology and chemical signalling, and likely play a critical role in shaping the ecological dynamics of carnivorous plants (Holopainen and Gershenzon 2010; Mäki et al. 2019). Variations in rainfall can affect water status, turgor, and metabolic activity, which in turn modulate the production and release of VOCs. During periods of increased rainfall, higher leaf water content and humidity may dilute volatile concentrations, potentially reducing their effectiveness in attracting prey over long distances (Horner et al. 2018). In contrast, drier conditions can induce mild water stress, concentrating VOC emissions and enhancing their signalling potency, which may be particularly advantageous in nutrient-poor habitats where insect availability is limited (Kesselmeier et al. 2002). Despite the recognized influence of precipitation on plant physiology and VOC dynamics, few studies have examined how variation in rainfall simultaneously affects volatile emissions and prey capture efficiency in carnivorous plants.

To address this knowledge gap, we conducted a study under both field and greenhouse conditions using *Drosera rotundifolia*, a widely distributed carnivorous plant species in Europe, sourced from two climatically distinct sites in north-western Spain: Serra do Cando (Galicia, Spain) and Serra de Ancares (León, Spain). Serra do Cando is characterized by high precipitation levels and a cool Atlantic climate, while Serra de Ancares experiences lower rainfall, greater thermal variability, and a Mediterranean influence. In each region, we estimated the number of preys captured and measured VOCs and SVOCs emissions from *D. rotundifolia* plants across seven populations (four in Serra do Cando and three in Serra de Ancares). We predicted that plants in the wetter, cooler Serra do Cando exhibit lower volatile emissions due to increased humidity, whereas plants from the drier, more thermally variable Serra de Ancares produce more concentrated volatile emissions to attract prey under harsher conditions. By linking climatic variation to volatile emissions and prey capture, this study advances our understanding of how carnivorous plants integrate environmental cues into adaptive foraging strategies.

## Materials and Methods

### Natural History

*Drosera rotundifolia* L. (Droseraceae), commonly known as the round-leaved sundew, is a perennial carnivorous plant with a broad distribution across the Holarctic region (Fleischmann 2021; Mohn et al. 2023). In Eurasia, its range extends from the Mediterranean mountain ranges of Portugal, Spain, Italy, and Corsica to the Balkan Peninsula, the Caucasus, Mongolia, South Korea, and Japan (Baranyai and Joosten 2016). The species thrives primarily in acidic, nutrient-poor environments such as bogs and peatlands, where it is typically found growing on mats of *Sphagnum fuscum* (Schimp.) H. Klinggr., often competing with surrounding mosses for space and resources (Svensson 1995). This species exhibits a distinctive growth form, developing basal leaf rosettes from which one or more flowering stems may emerge. During the winter, *D. rotundifolia* enters a state of dormancy, surviving as a compact bud of tightly furled leaves, known as a *hibernaculum*, which remains just below the surface to withstand harsh conditions (Thorén et al. 2003). As a carnivorous plant, *D. rotundifolia* employs specialized glandular hairs on its leaves to capture prey. These hairs secrete a glistening, sugary mucilage that ensnares insects. Once trapped, the prey is enzymatically digested, allowing the plant to absorb essential nutrients such as nitrogen and phosphorus, which are often scarce in its natural environment (Matušíková et al. 2005). The chemical composition of *D. rotundifolia* is characterized by four major classes of bioactive compounds: naphthoquinones, flavonols, anthocyanidins, and anthocyanins (Egan and van der Kooy 2013). Naphthoquinones, such as plumbagin, contribute directly to the plant’s carnivorous function by aiding in prey digestion through their antimicrobial and proteolytic-enhancing properties (Dávila-Lara et al. 2021; Rahman-Soad et al. 2021). Flavonols and anthocyanidins are involved in photoprotection and oxidative stress mitigation, while anthocyanins give the plant its characteristic red pigmentation, which can attract insect prey (Martin-Eberhardt et al. 2025). Together, these metabolites not only facilitate nutrient acquisition but also provide chemical defence against herbivores and microbial pathogens, enhancing overall survival and fitness (Egan and van der Kooy 2013; Hatcher et al. 2020). Despite the considerable attention devoted to the study of its specialized metabolites, to date, no investigations have addressed the emission of volatiles in this species.

### Study Area

**Fig. 1:**
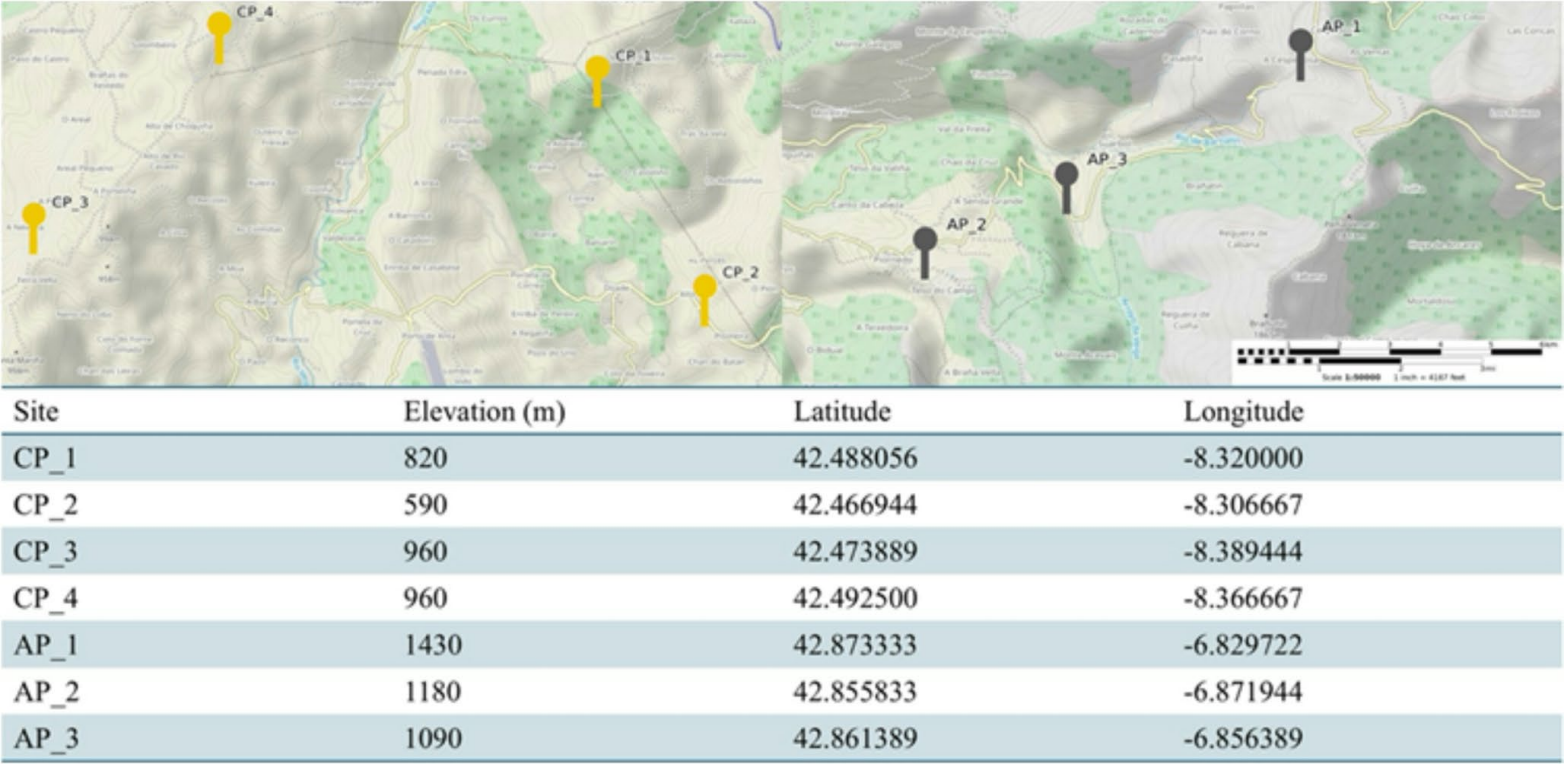
Map of the study sites showing populations of *Drosera rotundifolia* at two climatically distinct locations: Serra de Ancares (AP, drier site, grey) and Serra do Cando (CP, wetter site, yellow). Elevation, latitude, and longitude of each site are indicated

The study was conducted at two distinct sites in mainland Spain: Serra do Cando (CP), Pontevedra, Spain (42°28’09.3"N 8°23’08.0"W), and the Serra de Ancares mountain range (AP), León, Spain (42°51’18.7"N 6°52’20.7"W) (Fig. 1). At each site, populations of *D. rotundifolia* were identified, with four populations at CP and three at AP, each consisting of an average of 20 individuals. CP is a hilly region characterized by an Atlantic climate, which results in relatively stable temperatures and high annual precipitation. In 2024, the area received 234 L·m^-^² of rainfall (estación Rebordelo of MeteoGalicia, https://www.meteogalicia.gal/web/observacion/rede-meteoroloxica/historico). The landscape of CP is a mosaic of scrubland, rocky outcrops, and a mix of oak and riparian forests (Acuña-Alonso et al. 2024). In contrast, AP, situated within the Eurosiberian biogeographical region, is located near the Mediterranean zone and experiences a markedly different climate (Sánchez Vilas et al. 2025). It receives lower precipitation than the former site, with only 141 L·m^-^² recorded in 2024 (estación Ancares, MeteoGalicia). The plant communities in AP are characteristic of Atlantic, orophilous, and xeric-Mediterranean subtypes, reflecting the area’s more arid conditions and higher temperature variability (Ramil Rego et al. 2013). These two sites represent contrasting climatic and ecological conditions, providing valuable insights into the adaptability of *D. rotundifolia* in different environments across mainland Spain.

### Plant Sampling

Non-flowering *D. rotundifolia* plants were collected from the sampling sites in July 2024, ensuring that their root systems remained intact. A total of 10 individuals per population were sampled (*N* = 70). The plants were transported to the research-grade greenhouse at the Misión Biológica de Galicia-CSIC (Pontevedra, Spain) and carefully transplanted into pots containing a substrate mixture of commercial peat and perlite (4:1). The substrate was kept constantly moist to ensure optimal growth conditions. After a 20-day acclimation period, the plants were removed from the substrate, flash-frozen in liquid nitrogen, and stored at − 80 °C for subsequent chemical analyses.

### Prey Capture Assessment

For each plant, all fully opened and functional trap leaves were carefully harvested in the laboratory, excluding any that appeared senescent or necrotic. Captured insect prey were counted using a Nikon SMZ 745 T stereomicroscope (Nikon Corporation, Tokyo, Japan). Only complete insect bodies were recorded, while detached body parts (e.g., legs, antennae, wings) were excluded to prevent overestimation.

### Collection and Quantification of VOCs and Traps SVOCs

To analyse compounds emitted by *D. rotundifolia* trapping parts, we targeted both VOCs and SVOCs. VOCs are highly volatile molecules that readily evaporate and disperse, often mediating short-range ecological interactions such as prey attraction or signalling to mutualists and antagonists (Hare 2011; Jürgens et al. 2009; Turlings and Erb 2018). SVOCs are less volatile, more chemically stable compounds that can accumulate on plant surfaces and may contribute to constitutive defence or physiological stability (Ojeda et al. 2021). Capturing both classes provides complementary insights into the chemical ecology of the species.

We employed two extraction methods: solid-phase microextraction (SPME) for VOCs and hexane solvent extraction for SVOCs from the traps. All compounds were analysed using an Agilent 7890B gas chromatograph coupled with a 5977B mass selective detector, fitted with a 30 m × 0.25 mm × 0.25 μm HP-5MS fused silica column (Agilent, Santa Clara, CA, USA). For VOCs, entire leaf-stem sets, previously frozen at − 80 °C, were placed in 20 mL glass vials sealed with PTFE/silicone septa. A SPME fiber (Arrow 1.1 mm, 100 μm PDMS) was exposed to the headspace for 12 min at 35 °C with 250 rpm agitation to adsorb volatiles. The fiber was then thermally desorbed into the GC inlet at 250 °C for 3.5 min in splitless mode (13.5 psi, helium flow 1.0 mL·min⁻¹). The oven program started at 45 °C (3 min), ramped at 6 °C·min⁻¹ to 260 °C (1 min hold), followed by a 270 °C post run (2 min). VOCs were quantified as relative abundances based on mass spectrometer detection, without absolute quantification. For SVOCs, the entire aerial part of each plant was rinsed with 1.5 mL analytical-grade hexane (Fischer), with 200 ng nonyl acetate in 10 µL hexane added as an internal standard. Extracts were vortexed for 1 min, filtered, and 150 µL aliquots placed in vials. A 1.5 µL portion was injected into the GC inlet at 250 °C under pulsed splitless mode (7.05 psi). The oven program held at 60 °C (2 min), ramped at 6 °C·min⁻¹ to 270 °C (1 min hold, helium flow 0.9 mL·min⁻¹), followed by a 300 °C post run (2 min, helium flow 1.3 mL·min⁻¹). In all cases, the transfer line temperature was set to 280 °C for the mass spectrometry detector operating in electron impact mode (EI) at an ionization potential of 70 eV with a scan range of 33–550 (m/z), a source temperature of 230 °C, and a quadrupole temperature of 150 °C. Identifications were carried out by comparing experimental mass spectra with the references provided by the NIST 17 mass spectra library (US Department of Commerce). For both VOCs and SVOCs, Kováts indices were additionally calculated relative to the retention times of a n-alkanes solution (C8-C20, Sigma-Aldrich), obtained under identical analytical conditions to support compound identification further.

### Statistical Analyses

We tested the effect of source site (two levels: Serra de Ancares vs. Serra do Cando) on three response variables: (i) the total number of insect prey captured per plant; (ii) total VOC and SVOC emissions; and (iii) emissions of specific chemical classes, including sesquiterpenes, sesquiterpenoids, non-terpene derivatives and other compounds (unidentified naphtoquinone) for VOCs, and hydrocarbons, fatty acids, aldehydes, and other compounds—for example, decane, dodecane, tetradecane, hexadecane, an unidentified naphthoquinone, 2′-hydroxy-5′-methylacetoacetophenone, and methyl palmitate—for SVOCs. Source site was treated as a fixed factor, and population nested within site was included as a random factor to account for population-level variability. For insect prey abundance and VOC data, we fitted generalized linear mixed-effects models (GLMMs) with a Poisson error distribution and a log-link function. The number of trap leaves per plant was included as a covariate in the prey abundance model. For SVOC data, we used linear mixed-effects models (LMMs) with the same random structure, after log-transforming the data to improve residual normality.

To analyse VOC and SVOC composition, we performed permutational multivariate analyses of variance (PERMANOVA) with 9,999 permutations, based on Bray–Curtis dissimilarities of compound relative abundances. Population was included as a stratification factor to account for the nested sampling design (Anderson 2001). Differences in chemical composition between sites were visualized using Partial Least Squares Discriminant Analysis (PLS-DA). For each analysis, the six compounds with the highest loading magnitudes—calculated as the square root of the sum of squared loadings on components 1 and 2—were selected and displayed as vectors, highlighting the compounds that contributed most strongly to the separation between sites (Barker and Rayens 2003; Lê Cao et al. 2011).

All statistical analyses were performed in R version 4.5.1 (R Core Team 2025). GLMMs and LMMs were fitted using the *glmer* and *lmer* functions in the *lmerTest* package (Kuznetsova et al. 2017). The significance of fixed effects was assessed by likelihood-ratio chi-square tests (*Anova* function, *car* package) (Fox et al. 2013) for GLMMs and by F-tests (*standard anova()* function in *lmerTest* package) for LMMs. Post-hoc pairwise comparisons were conducted using Sidak tests with Kenward–Roger approximation for degrees of freedom (*lsmeans* function, *lsmeans* package) (Lenth 2016). PERMANOVA analyses were performed using the *adonis2* function in the *vegan* package (Oksanen et al. 2016), while PLS-DA analyses were conducted with the *plsda* function in the *mixOmics* package (Rohart et al. 2017). Graphs were built with *ggplot2* (Wickham 2016), *ggpubr* (Kassambara 2023), and *ggrepel* (Slowikowski 2016). Bar plots present back-transformed least-squares means with their standard errors, whereas PLS-DA plots display site centroids with 95% confidence ellipses and compound loading vectors to illustrate the variables contributing most strongly to group separation.

## Results

### Site Effects on Prey Capture Abundance

**Fig. 2:**
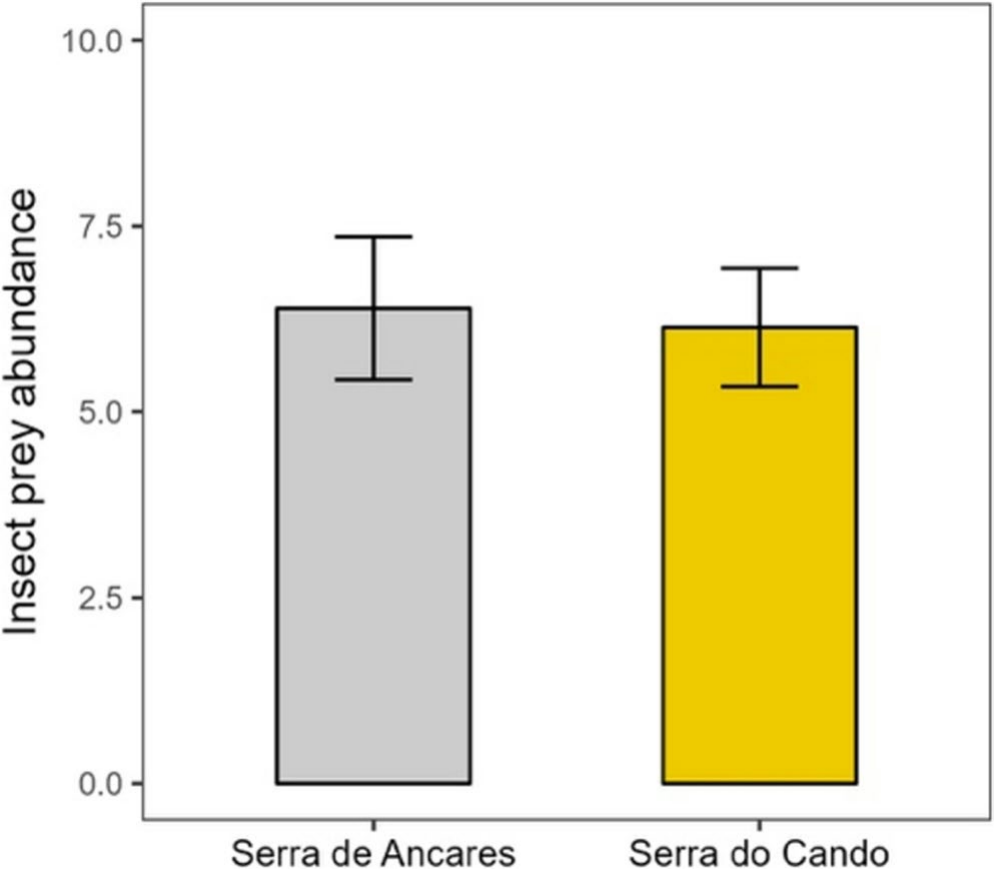
Mean insect prey abundance per plant in *Drosera rotundifolia* from the two study sites: Serra de Ancares (drier site, grey bar) and Serra do Cando (wetter site, yellow bar). Bars represent least-squares means ± SE (*N* = 27 for Serra de Ancares; *N* = 36 for Serra do Cando)

Prey capture abundance did not significantly differ between sites (Table 1). *Drosera rotundifolia* individuals from Serra de Ancares (AP) captured an average of 6.39 ± 0.96 insects per trap, while those from Serra do Cando (CP) captured 6.13 ± 0.80 insects per trap, indicating similar foraging success between the two populations in their natural habitats (Fig. 2).

**Table 1:** Effects of source site (two levels: the drier site in Serra de Ancares vs. the wetter site in Serra do Cando) were tested on multiple response variables: (i) total number of insect prey captured; (ii) total emissions of volatile organic compounds (VOCs) and semi-volatile organic compounds (SVOCs); (iii) emissions of specific chemical classes—sesquiterpenes, sesquiterpenoids, non-terpene derivatives, terpenoids, and fatty acids for VOCs, alkanes, green leaf volatiles, fatty acids, aldehydes, alcohols, ketones, terpenoid, and an unidentified naphthoquinone for SVOCs. Generalized Linear Mixed Models (GLMMs) and Linear Mixed Models (LMMs) were applied, with population included as a random effect in all models. SVOC data were log-transformed to meet normality assumptions. For PERMANOVA analyses, stratification by population was used to account for spatial structure

Response variable	Model family	Statistical test	df	*P*-value
**Prey abundance**	GLMM (Poisson)	χ^2^ = 0.042	1	0.839
**VOC emission**				
Total volatiles	GLMM (Poisson)	χ^2^ = 215.09	1	**< 0.001**
Sesquiterpenes	GLMM (Poisson)	χ^2^ = 1.37 × 10^8^	1	**< 0.001**
Sesquiterpenoids	GLMM (Poisson)	χ^2^ = 7.57 × 10^7^	1	**< 0.001**
Non-terpene derivatives	GLMM (Poisson)	χ^2^ = 8.72 × 10^5^	1	**< 0.001**
Terpenoids	GLMM (Poisson)	χ^2^ = 1.94 × 10^6^	1	**< 0.001**
Fatty acids	GLMM (Poisson)	χ^2^ = 5.37 × 10^6^	1	**< 0.001**
Other compounds	GLMM (Poisson)	χ^2^ = 8.59 × 10^8^	1	**< 0.001**
**SVOC emission**				
Total semi-volatiles	LMM (Gaussian)	F = 1.31	1	0.259
Alkanes	LMM (Gaussian)	F = 1.25	1	0.270
Green leaf volatiles	LMM (Gaussian)	F = 0.48	1	0.489
Fatty acids	LMM (Gaussian)	F = 0.68	1	0.414
Aldehydes	LMM (Gaussian)	F = 0.05	1	0.818
Alcohols	LMM (Gaussian)	F = 2.21	1	0.138
Ketones	LMM (Gaussian)	F = 4.60	1	**0.032**
Terpenoid	LMM (Gaussian)	F = 11.18	1	**< 0.001**
Other compounds	LMM (Gaussian)	F = 0.04	1	0.846
**Chemical composition**				
VOC composition	PERMANOVA	F = 2.82	1	1
SVOC composition	PERMANOVA	F = 1.07	1	1

Significant *P*-values (*P* < 0.05) are shown in bold

### Site Effects On the Emission and Composition of Volatile and Semi-volatile Compounds

**Fig. 3:**
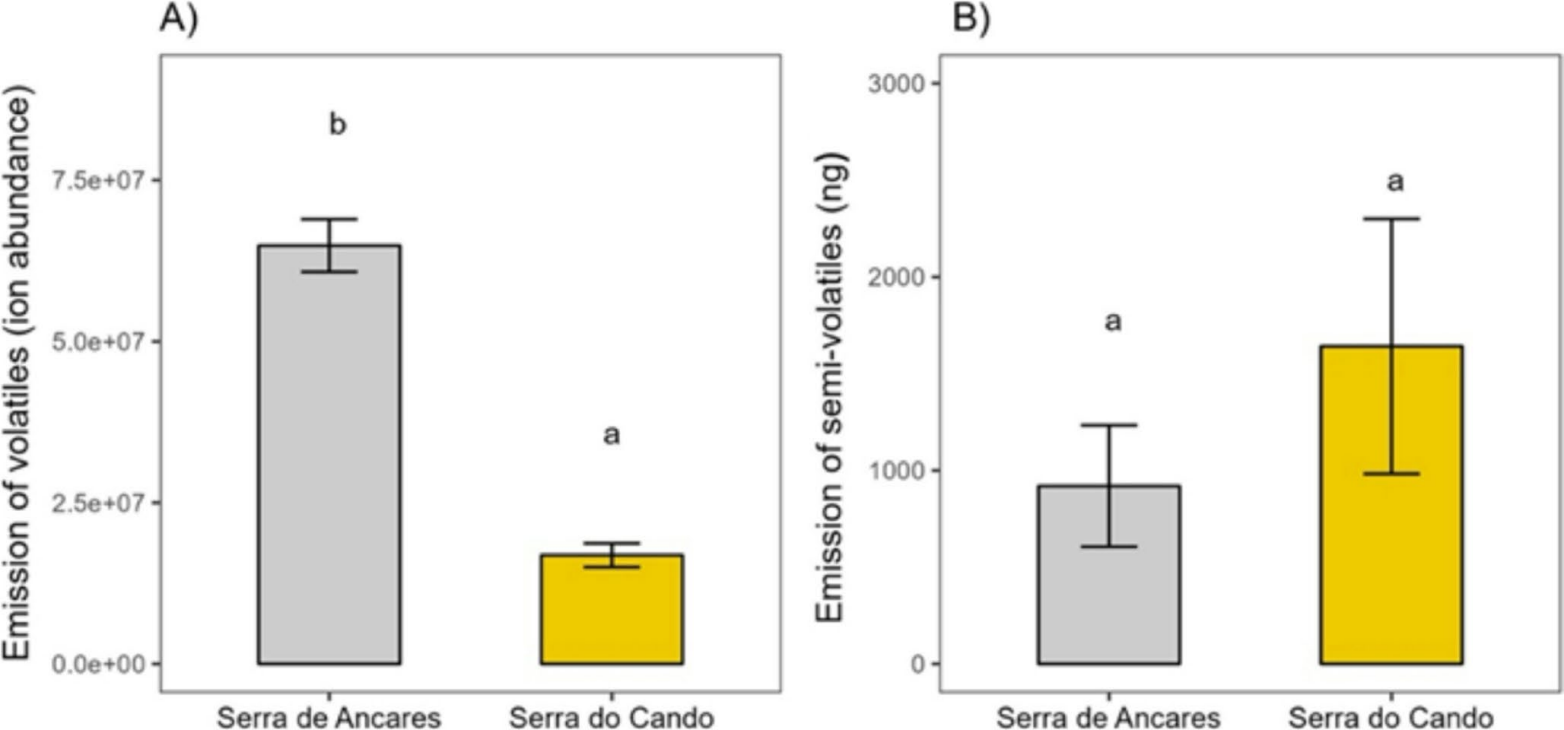
Total emission of volatile and semi-volatile organic compounds in *Drosera rotundifolia* sourced from Serra de Ancares (lower precipitation, grey bars) and Serra do Cando (higher precipitation, yellow bars) and grown under standardized greenhouse conditions. (A) Total volatile organic compound (VOC) emission, expressed as ion abundance. (B) Total semi-volatile organic compound (SVOC) emission, expressed as ng. Bars represent least-squares means ± SE (*N* = 24 for Serra de Ancares; *N* = 15 for Serra do Cando). Different letters indicate significant differences (*P* < 0.05)

**Fig. 4:**
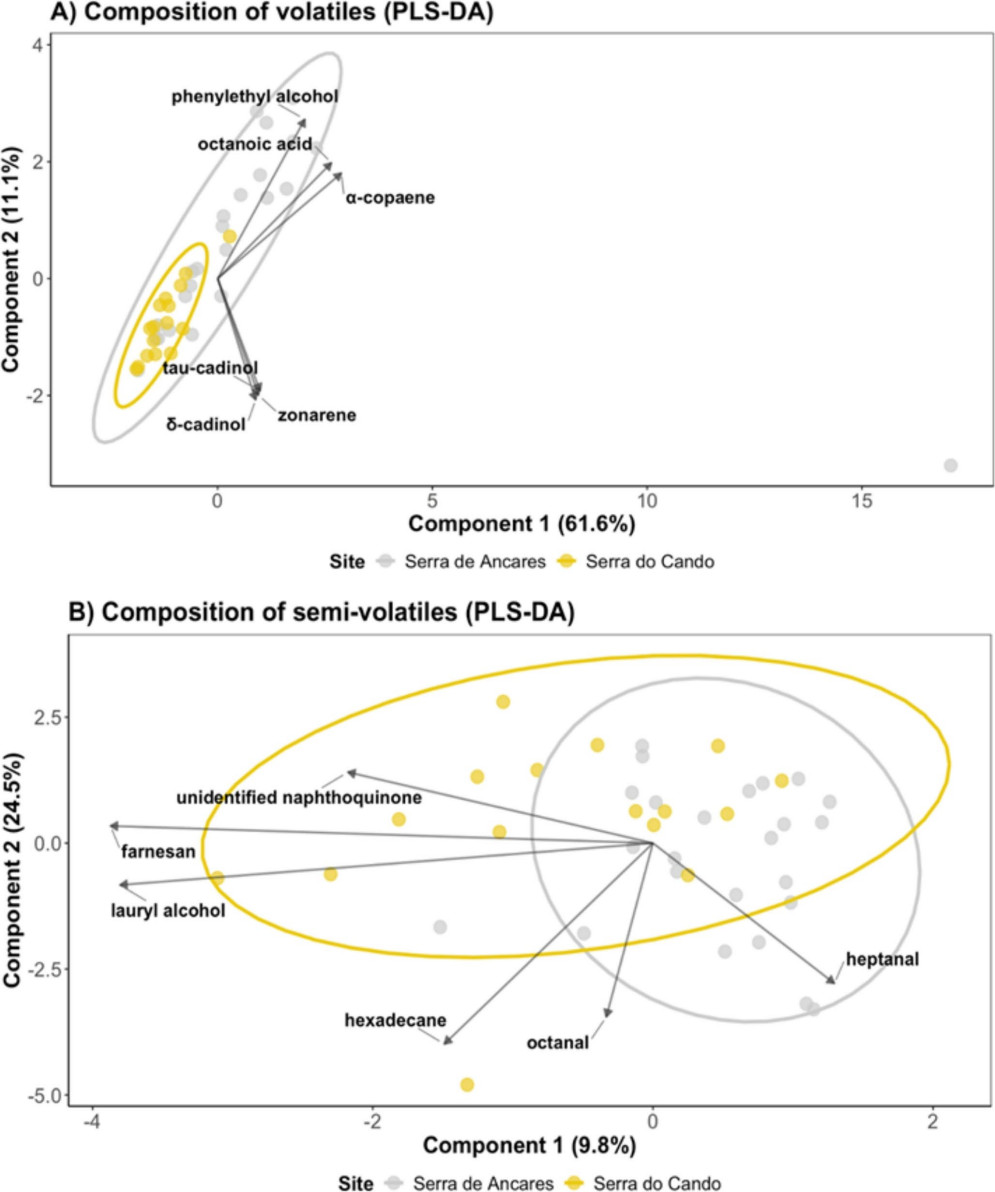
Partial Least Squares Discriminant Analysis (PLS-DA) of VOC and SVOC composition in *Drosera rotundifolia* from Serra de Ancares (drier site, grey dots, *N* = 24) and Serra do Cando (wetter site, yellow dots, *N* = 15). (**A**) VOC composition showing clear separation between sites (Component 1: 61.6%, Component 2: 11.1%). (**B**) SVOC composition (Component 1: 9.8%, Component 2: 24.5%). Ellipses represent 95% confidence intervals around site centroids, and arrows indicate compounds with the highest loadings that significantly contribute to site discrimination. Statistical analyses were performed at the chemical class level (see Table 1)

After 20 days of acclimation under standardized greenhouse conditions, total VOC emissions, as well as emissions of sesquiterpenes, sesquiterpenoids, non-terpene derivatives, terpenoids, and fatty acids were significantly higher in AP plants (the lower-precipitation site) compared to CP plants (Table 1; Fig. 3A). Three sesquiterpenes—α-copaene, longifolene, and cadina-3,5-diene—were detected exclusively in AP individuals, while an unidentified naphthoquinone was the most abundant compound overall (Table 2). In contrast, total SVOC emissions—including alkanes, green leaf volatiles, fatty acids, aldehydes, alcohols, ketones, terpenoid and an unidentified naphthoquinone—did not differ significantly between sites (Table 1; Fig. 3B), with the unidentified naphthoquinone remaining the dominant compound in both populations (Table 3). PERMANOVA analyses indicated that site had no significant effect on overall VOC or SVOC composition (Table 1), which was further supported by PLS-DA ordination showing overlapping VOC and SVOC profiles between sites (Fig. 4).

**Table 2:** Mean ion abundance of volatile organic compounds (VOCs) emitted by *Drosera rotundifolia* individuals from the wetter site, Serra do Cando (CP), and the drier site, Serra de Ancares (AP), grown under standardized greenhouse conditions

Compound		Class		Mean (Ion abundance)		
				CP		AP
α-copaene		Sesquiterpenes		0		441753.79 ± 127827.1
β-elemene		Sesquiterpenes		40381.87 ± 25,920. 7		1192914.6 ± 551640.1
β-gurjunene		Sesquiterpenes		47028.6 ± 24665.1		661,132 ± 401658.5
δ-cadinene		Sesquiterpenes		173945.1 ± 68505.3		3,820,979 ± 3335049.8
γ-muurolene		Sesquiterpenes		215856.6 ± 144978.4		3320206.8 ± 2311600.6
Cadina-3,5-diene		Sesquiterpenes		0		1348007.8 ± 1023375.9
Cis-muurola-4(15),5-diene		Sesquiterpenes		307233.2 ± 126702.9		3731440.6 ± 2746252.5
Cubenene		Sesquiterpenes		94274.8 ± 44024.2		3006162.8 ± 1683814.7
Longifolene		Sesquiterpenes		0		226986.58 ± 207035.1
Zonarene		Sesquiterpenes		55712.13 ± 52230.1		5142432.1 ± 4862847.0
δ-cadinol		Sesquiterpenoids		343593.4 ± 136544.1		1,145,564 ± 841097.4
Epicubenol		Sesquiterpenoids		1,708,546 ± 463293.5		4224958.7 ± 1087219.9
Tau-cadinol		Sesquiterpenoids		248689.6 ± 99422.0		6500122.1 ± 5750128.0
Cis-ligustilide		Non-terpene derivatives		3,121,280 ± 1963910.6		4867449.9 ± 1733327.8
Phenylethyl alcohol		Non-terpene derivatives		21302.47 ± 19971.1		799599.92 ± 336346.8
Eucalyptol		Terpenoids		1,758,569 ± 461264.0		2236257.7 ± 310161.6
Hexahydrofarnesyl acetone		Terpenoids		290115.3 ± 151971.3		507590.67 ± 134908.3
Octanoic acid		Fatty acids		249775.4 ± 115750.9		1101015.3 ± 266486.6
Unidentified naphthoquinone		Other compounds		14,191,386 ± 5490336.8		138,334,666 ± 72162269.5

Values represent average ion abundance per compound, calculated across individuals within each site. Statistical analyses were performed at the chemical class level (see Table 1)

**Table 3:** Mean emission (ng) of semi-volatile organic compounds (SVOCs) detected in *Drosera rotundifolia* individuals from the wetter site, Serra do Cando (CP), and the drier site, Serra de Ancares (AP), grown under standardized greenhouse conditions

Compound	Class	Mean (ng)	
		CP	AP
Decane	Alkanes	7.6 ± 0.8	8.9 ± 0.8
Dodecane	Alkanes	8 ± 0.8	6.3 ± 1.5
Hexadecane	Alkanes	1.5 ± 0.1	0.5 ± 0.3
Tetradecane	Alkanes	0.3 ± 0.0	0.3 ± 0.1
2-hexanol	Green leaf volatile	17.9 ± 1.5	20 ± 10.3
3-hexanol	Green leaf volatile	33.3 ± 2.2	27.2 ± 13.5
n-hexadecanoic acid	Fatty acids	126.5 ± 150.7	222.2 ± 122.5
Linoleic acid	Fatty acids	153.3 ± 171.3	258.9 ± 133.0
Methyl palmitate	Fatty acids	2.1 ± 1.5	2.5 ± 1.2
Heptanal	Aldehydes	4.2 ± 0.9	5.3 ± 0.8
Octanal	Aldehydes	3.7 ± 0.8	3.5 ± 0.8
Lauryl alcohol	Alcohols	2.5 ± 0.2	0.9 ± 0.3
2’-hydroxy-5’-methylacetoacetophenone	Ketones	253 ± 12.8	115.8 ± 46.6
Farnesan	Terpenoid	6 ± 0.2	0 ± 0.2
Unidentified naphthoquinone	Other compounds	2491.2 ± 413.8	1713.5 ± 404.6

Values represent average emissions per compound across individuals within each site. Statistical analyses were performed at the chemical class level (see Table 1)

## Discussion

Our results show that prey capture by *D. rotundifolia* was similar at both climatically contrasting sites, indicating consistent foraging success across environments. After acclimation under greenhouse conditions, plants from the drier AP site emitted higher total amounts of VOCs, particularly sesquiterpenes, but showed no increase in SVOC emissions or major changes in VOC and SVOC composition. Collectively, these findings suggest that carnivorous plants, depending on the local ecological conditions, can adjust the intensity of volatile emissions and exhibit structured variation in VOC profiles without substantial shifts in overall chemical diversity, while semi-volatile profiles remain relatively stable.

We found that *D. rotundifolia* populations from AP and CP exhibited similar prey capture success and trapping efficiency despite pronounced environmental differences. This contrasts with previous studies showing that prey availability and capture rates in carnivorous plants often increase in shadier, more humid, and more fertile habitats (e.g., Alcalá and Domínguez 2003; Cook et al. 2018). One explanation for this discrepancy is that earlier work often examined performance along strong gradients of resource availability, or light, within single regions. Instead, our comparison involved populations that may experience different climatic conditions but similar nutrient limitations, maintaining comparable selective pressures on foraging efficiency. In addition, prey capture in *D. rotundifolia* may be constrained by conserved structural and mechanical traits, such as trap size and mucilage properties, which limit variation in capture efficiency (Wheeler and Carstens 2018). Importantly, prey capture was recorded after plants from AP and CP had been housed together in a greenhouse for 20 days, meaning the counted insects likely reflect a more homogenous prey availability landscape, rather than natural differences between regions. Consequently, the observed similarities may not reflect in situ performance, and caution is warranted in interpreting these results in an ecological context.

In contrast to prey capture, VOC emissions differed between populations grown under common greenhouse conditions, with AP plants producing higher levels of VOCs. Similar increases in volatile production under water limitation have been widely documented in other species, supporting the idea that drought-related stress is closely linked to enhanced synthesis or release of specialized metabolites (e.g., Holopainen and Gershenzon 2010; Rissanen et al. 2022). Because our plants were analysed under standardized conditions, the observed differences indicate that population-level variation in VOC production can persist independently of immediate environmental effects. At the same time, previous field-based studies have shown that VOC emissions are strongly modulated by short-term environmental factors, including temperature, radiation, and plant water status (reviewed by Aartsma et al. 2017; Holopainen and Gershenzon 2010). Under natural conditions, these drivers can substantially alter both emission rates and compound profiles. Consequently, measurements conducted in the field would probably reflect a stronger influence of microclimate and temporal variability, whereas our greenhouse experiment isolates underlying differences among populations.

Our results showed that total SVOC emissions did not differ significantly between the drier AP and wetter CP sites, indicating that SVOC production is relatively stable across environmental gradients. To our best knowledge, this is the first study examining SVOC emissions across contrasting habitats in this species, providing new insight into the consistency of plant specialized metabolites under variable conditions. This stability likely reflects the essential physiological roles of SVOCs, including membrane stabilization and serving as precursors for other specialized metabolites, which impose strong functional constraints on their production (Chen et al. 2011; Yang et al. 2025). Unlike VOCs, SVOCs appear less responsive to short-term environmental variation, suggesting limited phenotypic plasticity and potential genetic constraints (Ivey et al. 2009; Valladares et al. 2007). These findings suggest that SVOCs constitute a relatively stable and conserved component of *D. rotundifolia*’s chemical profile, complementing the more variable VOC fraction.

We found no relationship between VOC emissions and trap efficiency, indicating that volatile production and prey capture are not tightly coupled. This contrasts with previous work reporting that higher VOC emissions increase insect abundance and influence prey composition in carnivorous plants (e.g., Dupont et al. 2023; Jürgens et al. 2009). One reason for this difference is that trap efficiency in *Drosera* depends primarily on structural and mechanical traits, including trap size, shape, and mucilage stickiness (Horner et al. 2018), which impose strong functional constraints on prey capture regardless of volatile output. Additionally, prey behaviour and environmental context can strongly influence capture success, further decoupling VOC production from trapping. Volatile compounds likely serve multiple ecological functions beyond prey attraction, including defence against herbivores and microbial pathogens and signalling (Heil 2014; Ninkovic et al. 2021), which may dilute any effect on trap efficiency. Together, these factors help explain why elevated VOC emissions in our study did not translate into higher prey capture, highlighting the complex and multifaceted role of volatiles in carnivorous plant ecology.

Overall, our results show that *D. rotundifolia* maintains consistent prey capture across contrasting environments, despite population-level variation in VOC emissions and stable SVOC profiles, highlighting a decoupling between chemical plasticity and functional foraging traits. Future research should integrate field-based measurements of VOC and SVOC emissions with ecological performance to evaluate how environmental variability shapes population-level chemical traits. In situ experiments would capture short-term effects of temperature, light, humidity, and water availability on VOC production, potentially revealing more nuanced differences among populations than those observed under standardized greenhouse conditions. Bioassays with representative prey and natural enemies could clarify the functional role of VOC variation in prey attraction and indirect defence, while SVOC analyses could assess contributions to constitutive protection and physiological stability. Combining metabolomic profiling with genetic and transcriptomic approaches could uncover the molecular basis of volatile biosynthesis and determine whether observed differences reflect adaptive evolution or phenotypic plasticity, ultimately enhancing understanding of how carnivorous plants balance chemical investment with foraging efficiency under variable environments.

## Supplementary Information

Below is the link to the electronic supplementary material.

Supplementary Material 1 (XLSX 19.7 KB)

## Data Availability

Not applicable.
